# Small Molecule Optoacoustic Contrast Agents: An Unexplored Avenue for Enhancing In Vivo Imaging

**DOI:** 10.3390/molecules23112766

**Published:** 2018-10-25

**Authors:** Matt D. Laramie, Mary K. Smith, Fahad Marmarchi, Lacey R. McNally, Maged Henary

**Affiliations:** 1 Department of Chemistry, 100 Piedmont Avenue SE, Georgia State University, Atlanta, GA 30303, USA; mlaramie1@student.gsu.edu (M.D.L.); fjrjes1@student.gsu.edu (F.M.); 2 Center for Diagnostics and Therapeutics, 100 Piedmont Avenue SE, Georgia State University, Atlanta, GA 30303, USA; 3 Department of Cancer Biology, 1 Medical Center Blvd, Wake Forest Comprehensive Cancer Center, Winston-Salem, NC 27157, USA; mksmith@wakehealth.edu

**Keywords:** optoacoustic, near-infrared, small molecule, contrast agents

## Abstract

Almost every variety of medical imaging technique relies heavily on exogenous contrast agents to generate high-resolution images of biological structures. Organic small molecule contrast agents, in particular, are well suited for biomedical imaging applications due to their favorable biocompatibility and amenability to structural modification. PET/SPECT, MRI, and fluorescence imaging all have a large host of small molecule contrast agents developed for them, and there exists an academic understanding of how these compounds can be developed. Optoacoustic imaging is a relatively newer imaging technique and, as such, lacks well-established small molecule contrast agents; many of the contrast agents used are the same ones which have found use in fluorescence imaging applications. Many commonly-used fluorescent dyes have found successful application in optoacoustic imaging, but others generate no detectable signal. Moreover, the structural features that either enable a molecule to generate a detectable optoacoustic signal or prevent it from doing so are poorly understood, so design of new contrast agents lacks direction. This review aims to compile the small molecule optoacoustic contrast agents that have been successfully employed in the literature to bridge the information gap between molecular design and optoacoustic signal generation. The information contained within will help to provide direction for the future synthesis of optoacoustic contrast agents.

## 1. Introduction

### 1.1. MSOT Introduction:

Visualization of fluorescent dyes is highly limited to superficial levels, as the scattering of light degrades spatial resolution at increased depths [1]. While the development of near-infrared dyes has increased the depth of fluorescent detection, the visualization of these dyes remains limited to a few millimeters. Emerging technologies, such as fluorescence molecular tomography, have improved this to depths of up to 10 cm, the instrumentation for this has not yet reached a clinically-relevant size, and cannot be used to provide real-time guidance [2,3,4]. To overcome these limitations of detection depth, multispectral optoacoustic tomography (MSOT) has emerged as an alternative modality that relies on the optoacoustic effect in which molecules within biological tissues absorb light, causing them to heat up and expand. This thermoelastic expansion generates ultrasonic waves which can be detected and converted to an image [5]. Because most tissues are relatively transparent to light in the range of 650 to 900 nm, MSOT uses NIR light to excite molecules which generate soundwaves. These ultrasonic waves are considerably less scattered in tissue than photons, which allows for deeper tissue penetration (>3 cm) with higher accuracy and resolution than purely optical imaging [6,7]. While there are some endogenous contrast agents used in optoacoustic imaging, the use of exogenous contrast agents such as small molecule dyes is necessary for a wider range of biological activities including acidosis [8,9] and ion trafficking [10,11] to be observed and monitored. This is especially evident when it is taken into account that MSOT can identify and delineate multiple contrast agents in a single sample/image. While fluorescence imaging requires fluorophores to emit light of distinct wavelengths with minimal spectral overlap to resolve separate signals, MSOT is able to resolve contrast agents with similar absorption profiles. Unlike fluorescence imaging, which records single wavelengths and requires contrast to fluoresce at distinct wavelengths, MSOT records the entire optoacoustic spectral profile; by using the entire spectrum, different contrast agents can be resolved based on the overall shape of the spectrum.

Note that while we use the term “optoacoustic” here, the terms “photoacoustic” and “optoacoustic” are used interchangeably in the literature. The deciding factor in preference for either of the two seems to be geographical, with North America largely using “photoacoustic”, while Europe prefers the term “optoacoustic”.

### 1.2. Exogenous Contrast for Optoacoustic Imaging

Exogenous contrast agents for optoacoustic imaging consist of three main classes of compounds: organic dyes, nanoparticles, and organic polymers. Each class of contrast offers differing strengths and weaknesses for in vivo imaging. Nanoparticles are highly tunable, which is their greatest strength and weakness. Small changes in the shape or size of nanoparticles can vastly change their optical properties, biodistribution, or toxicological profile [12,13]. Retention in the liver, spleen, and bone marrow remains problematic for nanoparticles [12]. Often, tumor uptake of nanoparticles is only by enhanced permeability and retention (EPR) effect, which results in only limited accumulation of contrast in cancerous tissue, and has failed to translate to humans in clinical trials, although surface modification with targeting peptides or antibodies can improve tumor uptake [14]. Semiconducting organic polymers, like nanoparticles, rely heavily on EPR for tumor uptake, but, unlike nanoparticles, are more difficult to functionalize with targeting ligands [15,16,17]. Fluorescent proteins have also been successfully applied in optoacoustic imaging, allowing researchers to monitor genetically-encoded contrast [18,19,20]. All of these classes have already been thoroughly reviewed in the current literature. Organic dyes have extremely appealing properties for use in optoacoustic imaging; however, they have not received as much attention as the other two classes of contrast agents. While novel nanoparticles and semiconducting polymers have been reported in optoacoustic applications, the use of organic dyes has been limited mostly to commercially-available dyes, which are poorly optimized for optoacoustic performance. Reviews of optoacoustic contrast agents, similarly, have overlooked the design of organic dyes, focusing instead on the design of molecular probes consisting of dyes and targeting agents or activating groups [21,22,23,24,25].

### 1.3. Near-Infrared Organic Dyes Introduction

There is a wide variety of small-molecule dyes that absorb light in the near-infrared (NIR) region, which makes them viable candidates for use as exogenous contrast agents for optoacoustic tomography. Many of these NIR dyes generate a detectable signal (fluorescent or acoustic) [26]. Multiple families of these dyes have been synthesized utilizing diverse scaffolds, including cyanines [27], bodipy [28], phthalocyanines [29], and perylenes [30,31,32]. NIR dyes have raised a great deal of interest due to their versatility in a wide array of applications, low energy absorption, and the synthetic modifiability of the small molecule organic contrast agents [33,34,35,36,37,38,39]. NIR contrast agents for fluorescence imaging have been widely explored. Many libraries of NIR dyes have been generated and screened, and correlations between physicochemical properties and function (biodistribution, quantum yield of fluorescence, etc.) have been generated for NIR fluorophores; however, no comparable effort has been expended for optoacoustic contrast agents [40]. It is well described that, for an optically absorbing body, the amplitude of the ultrasonic wave produced upon excitation will be described by:

P = Γ × μ_a_ × Φ (1)

where Γ is the Grüneisen parameter of the system, μ_a_ is the absorption coefficient of the absorber, and Φ is the fluence of the excitation light at the absorber [41,42,43]. The current, unmet need then is determining the relationship between the Grüneisen parameter and the organic dye structure. By determining what functional groups lead to more effective generation of optoacoustic signal, improved contrast agents can be developed for this powerful imaging technology.

This review focuses on widely-used, commercially-available NIR dyes that are commonly used in fluorescence imaging, and their potential for optoacoustic imaging. Physicochemical properties were calculated for each dye along with optoacoustic signal testing in phantoms to give insight into which properties of the NIR dye are important to yield an improved optoacoustic signal. There is a current unmet need for improved contrast agents in optoacoustic imaging; however, too little is known about what structural features of NIR dyes lead to effective optoacoustic signal generation. By illustrating the utility of common dyes typically used in fluorescence imaging in optoacoustic imaging while probing the structural features that enable these compounds to produce optoacoustic signals, we hope to engage both dye chemists and imaging specialists in answering the unmet need for improved contrast agents for optoacoustic imaging.

The contrast agents will be separated into three categories: dyes with absorbances below 720 nm, dyes with absorbances above 720 nm, and dark quenchers. The rationale behind these categories is due to the potential for multiplex imaging innate to optoacoustic imaging and the most common absorbance wavelengths of common organic fluorophores. Dyes from each of the different categories could be used simultaneously in a single co-injection to highlight separate anatomical structures. Dark quenchers have been separated out as they are a less studied class of dyes; their normal use is in Förster resonance energy transfer (FRET) -based applications, where they simply quench the fluorescence signal. However, as they are strongly absorbing and non-fluorescent, they may be applicable as optoacoustic probes in their own right.

## 2. Contrast Agents

### 2.1. Dyes with Absorbance < 720 nm

The majority of the dyes that absorb less than 720 nm are blue dyes. Blue dyes have a myriad of different biological applications. Specifically, they are often utilized as various biological stains due to the fact that they are often visible to the naked eye and are easily contrasted against common biological media such as blood. Because much of their biological activity has already been explored and reported, their potential to be used as in vivo exogenous contrast agents for optoacoustic imaging is elevated. The applications of blue dyes as optoacoustic contrast agents are often an extension of their already well-defined biological uses. Unfortunately, blue dyes absorb on the low end of the desired 650–900 NIR range utilized in MSOT. This results in spectral overlapping with endogenous contrast agents such as hemoglobin. Further, blue absorbance is on the edge of detection for most MSOT instrumentation.

#### 2.1.1. Evans Blue

Evans blue is an FDA-approved, non-toxic dye that has many biological applications, including use in the measurement of blood volume [44] and permeability of the blood-brain barrier [45]. Evans blue is a diazo-dye containing multiple conjugated aromatic rings. The inclusion of multiple sulfonate substituents renders the dye highly water soluble.



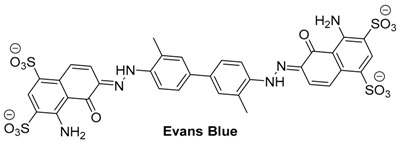



**Figure 1:**
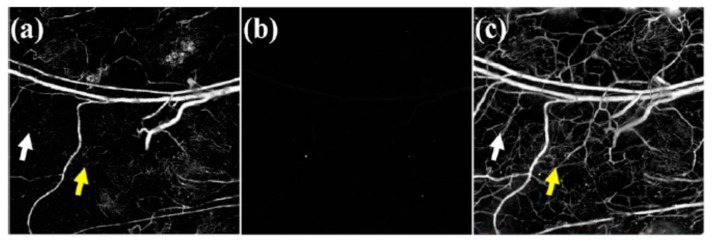
(**a**) Optoacoustic image of naked mouse ear before Evans blue injection at 570 nm (**b**) optoacoustic image of naked mouse ear before Evans blue injection at 610 nm. (**c**) optoacoustic image of naked mouse ear after Evans blue injection at 610 nm. Arrows indicated capillaries that become visible and continuous with Evans blue injection. Figure from [48] reproduced with permission from the publisher.

The combination of its ability to tightly bind to serum albumin with its strong absorption in NIR at 620 nm has made Evans blue an ideal contrast agent for the optoacoustic imaging of the microvascular network [46,47]. In 2009, Evans blue was used to image capillary networks, as well as to study the diffusion dynamics of the dye and clearance of the albumin-bound dye [48]. In the study, optoacoustic images of the microvessels of a mouse’s ear were acquired at both 570 nm and 610 nm. At 570 nm, hemoglobin has strong absorption, so there was high contrast and signal-to-noise ratio. Larger veins and arteries contained more red blood cells, so they were uniformly bright. However, smaller capillaries appeared discontinuous and broken (Figure 1a). As hemoglobin has very weak absorption at 610 nm, the optoacoustic signal was considerably weaker (Figure 1b). Then, a 6% solution of Evans blue was injected into the bloodstream of a nude mouse. Immediately after the injection of Evans blue, the network of small capillaries no longer looked fragmented, with many of the previously unseen smaller branching points becoming visible (Figure 1c). Evans blue has also been used as the contrast agent for the noninvasive optoacoustic imaging of the cortical vascular network in mice [49].

Unfortunately, the ability to bind albumin, which makes Evan’s blue ideal for vascular imaging, is also a major factor limiting its use as an optoacoustic contrast agent, as it prevents tissue specific uptake [50].

#### 2.1.2. Methylene Blue

Another FDA-approved blue dye that has been used in optoacoustic imaging is methylene blue. Methylene blue has been used for a broad range of medical applications since the early 19th century, when it was initially reported as a treatment for malaria [51]. In research settings, methylene blue is an extremely common biological and chemical stain most often used for bacteriology or as redox indicator. In terms of imaging, methylene blue is often utilized because it is easy to observe and monitor visually, without the need for specialized equipment; however, this use is, of course, limited to superficial levels or intraoperative procedures.



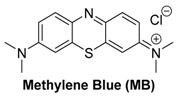



**Figure 2:**
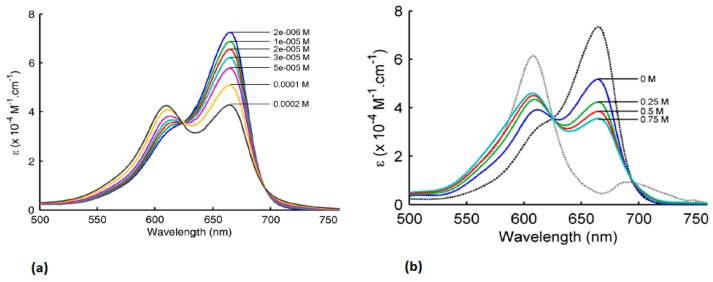
(**a**) Absorbance spectra of different concentrations of methylene blue in water (**b**) Absorbance spectra of 80 µM solution of methylene blue in water with 4 different salt concentrations (grey dotted line is absorbance of dimer in water, black dotted line is absorbance of monomer in water) Figure from [54] reproduced with permission from the publisher.

Methylene blue is a planar tricyclic phenothiazine which exists as a blue cation under physiological conditions. It is often commercially available as the hydrate. Methylene blue is highly water soluble, with a solubility of 50 milligrams per milliliter of water. Methylene blue is cleared through the kidneys, and is excreted either unchanged or as its metabolite leucomethylene blue [52,53]. Further, in solution, methylene blue can exist in either a monomeric or dimeric form, depending on its concentration or the salt concentration of the solvent, which can lead to a change in the absorbance spectrum (Figure 2) [54]. The monomeric form of methylene blue has a peak absorbance of 665 nm.

**Figure 3:**
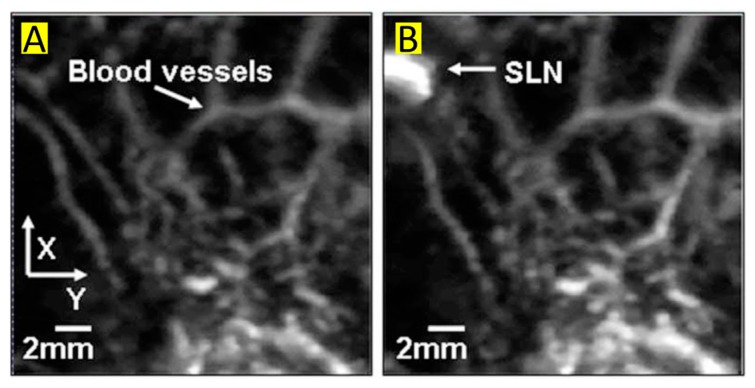
(**A**) optoacoustic image of control without methylene blue at 635 nm (**B**) optoacoustic image of SLN 52 min after injection of MB at 635 nm. Figure from [55] reproduced with permission from the publisher.

Methylene blue is the most utilized contrast agent for optoacoustic imaging, with a peak absorption less than 720 nm. One of the most discussed studies uses methylene blue as an exogenous agent for the optoacoustic detection of sentinel lymph nodes (SLN), a key procedure for the staging of early breast cancer. Early studies demonstrated the ability of optoacoustic systems to detect SLN in vivo at 635 nm in rats after an intradermal injection of 0.7 µL of a 3mM solution of methylene blue (Figure 3) [55].

Optoacoustic imaging of methylene blue-stained SLN was later combined with ultrasound imaging of the surrounding tissue [56]. The combination of optoacoustic with ultrasound imaging allows for easier, non-invasive identification of SLN and procurement of morphological information such as size and shape of the SLN. This technology has continued to be developed, and is currently being used in clinical studies. Optoacoustic imaging has allowed for the accurate identification of SLN stained with methylene blue (a 3mM solution injected subcutaneously into a human breast) in human patients [57]. The ultimate goal of this optoacoustic technology is to accurately guide percutaneous fine needle aspiration biopsy, leading to a far less invasive procedure for lymph node staging.

Methylene blue has also been combined with microbubbles in order to develop a contrast agent that was active for both optoacoustic and ultrasound imaging [58]. Interestingly, it was reported that while the intensity of the optoacoustic signal was significantly affected by the concentration of microbubbles, the intensity of the ultrasound signal was not similarly affected by the concentration of the methylene blue. Further, methylene blue has been utilized as an optoacoustic contrast agent to measure oxygen pressure in tumors [59], as well as encapsulated in nanoprobes in an effort to develop targeted optoacoustic imaging [60].

Though it is a commonly-employed contrast agent for optoacoustic imaging, methylene blue still has limitations. First, it is known for its relatively rapid body clearance; thus, is not ideal for extended imaging [60]. Further, enzymatic reduction of methylene blue to leucomethylene blue leads to the loss of the chromophore and of NIR absorption [61].

#### 2.1.3. Coomassie Blue

Coomassie blue (CB) is another blue NIR dye that is most commonly used for staining proteins in gel electrophoresis and Bradford-type assays for protein quantitation. In acidic conditions, Coomassie blue binds to proteins via the basic amino acids. Once bound, the dye becomes a bright blue color. Along with easy visual delineation, Coomassie blue is also compatible with mass spectrometry, and protein-bound CB has been shown to fluoresce in the 700–800 nm range, allowing for multiple modes of detection [62].



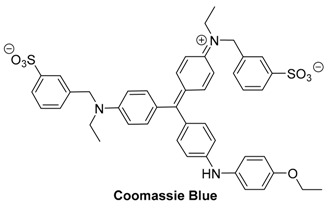



Coomassie blue is a triphenylmethane dye. As there is a lack of many solubility enhancing functional groups, Coomassie blue is largely insoluble in water. It absorbs in the near-IR region with a peak at 595 nm.

**Figure 4:**
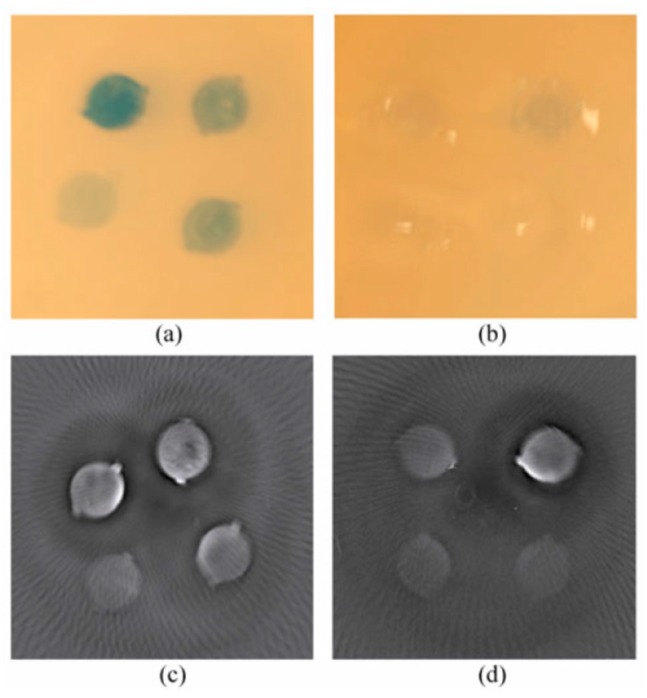
(**a**,**b**): photographs of phantoms with concentrations varying from 0.5 mg/mL (upper left corner of (**a**)) to 0.01 mg/mL (lower right corner of (**b**)) (**c**,**d**): optoacoustic images of the respective phantoms. Figure from [63] reproduced with permission from the publisher.

As a contrast agent for optoacoustic imaging, Coomassie blue has only been used in conjunction with nanoparticles. In 2011, optoacoustic imaging of F3 peptide-conjugated, hydrogel nanoparticles containing Coomassie blue was used to delineate brain tumors from normal tissue in rats [63]. The nanoparticle encapsulating Coomassie blue had previously been developed to selectively target brain tumors, and utilized to visually delineate brain tumors [64]. It was found that with the use of optoacoustic imaging, tumors treated with doses of nanoparticles that were too low to be visualized by the naked eye could still identified. The sensitivity of the optoacoustic system first became apparent with phantom experiments (Figure 4). Concentrations of nanoparticles which were 7% Coomassie blue by mass ranging from 0.5 mg/mL to 0.005 mg/mL were included into agarose phantoms, and examined by the optoacoustic system. While the higher concentrations were clearly visible to the naked eye, concentrations below 0.04 mg/mL were no longer distinguishable (Figure 4a,b). Alternatively, the optoacoustic system provided recognizable images for concentrations as low as 0.01 mg/mL, which is equivalent to 0.84 µM concentration of non-encapsulated Coomassie blue dye. Ex vivo studies on tumors in rat brains further supported that tumors containing low doses of the blue nanoparticles (125 mg/kg), while not visible to the naked eye, could be delineated from normal brain tissue using optoacoustic imaging.

Because Coomassie blue absorbs light around 595 nm, its spectrum often overlaps with the strong absorption from the blood. This overlap leads to high background signals in the imaging, significantly limiting the utility of Coomassie blue as a contrast agent for optoacoustic imaging. Further, its high affinity for binding to most proteins makes it unlikely to be used without being encapsulated in some capacity.

#### 2.1.4. Rhodamine Dyes

Rhodamine dyes are a broad class of fluorophores. Because of their excellent photophysical properties and photostability, they have been used for a variety of applications, including mitochondria staining [65] and flow cytometry [66]. Unfortunately, the majority of commercially-available rhodamine dyes absorb light which is significantly below the desired NIR range of 650–900 nm necessary for optoacoustic imaging.

**Figure 5:**
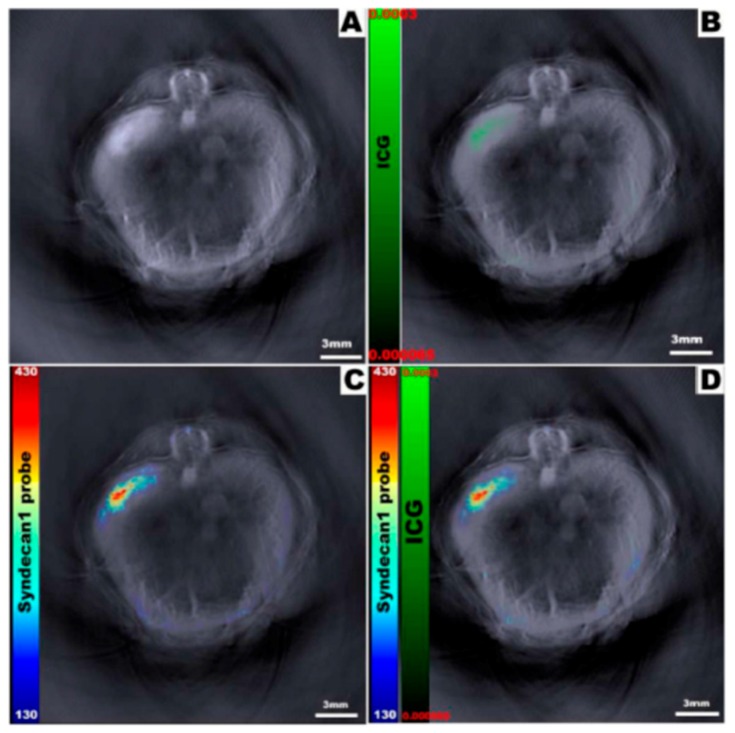
The ability to view multiple dyes in a single animal simultaneously is demonstrated. (**A**) no exogenous contrast agent (**B**) Image with only ICG (**C**) Image with only CF-750 probe (**D**) Image with both ICG and CF-750 Figure from [67] reproduced with permission from the publisher.

However, the commercially-available rhodamine dye, CF-750, the exact structure of which is proprietary, does absorb in the desired range, and has been used as a contrast agent for optoacoustic imaging [68]. In 2014, Hudson and Huang et al. conjugated CF-750 to epidermal growth factor (EGF) for use as an optoacoustically-active probe for upregulated EGF receptors in orthotopic pancreatic cancer [67]. The absorbance spectrum of CF-750 peaks at 750 nm, and was not significantly altered after conjugation to EGF. The degree of labeling of EGF with CF-750 was found to be 1.32, and mice were injected with a 100 nmol/L solution of the conjugated probe. The probe successfully targeted and accumulated in tumors within which EGF receptors were upregulated. MSOT was then able to non-invasively detect the probe-laden tumors at depths greater than 5 mm with great precision A year later, it was demonstrated that CF-750, when conjugated to a pancreatic cancer-targeting protein, could be distinguished from another contrast agent that was co-injected to the same animal model (Figure 5) [69]. This is one of the first examples in which two distinct exogenous contrast agents were distinguishable at depth via MSOT imaging.

Another commercially-available rhodamine dye which absorbs within the desired range for MSOT is Rhodamine 800, which boasts a peak absorption of 682 nm. Rhodamine 800 is, of course, comprised of the well-known xanthene core with two dialkylamino groups. The amine substituents are further incorporated into additional ring structures, increasing the rigidity of the structure. This rigidity, in combination with inclusion of the strongly-electron-withdrawing cyano group on the central carbon of the xanthene core, significantly shifts the absorbance of Rhodamine 800 into the ideal NIR region [70]. Unfortunately, the structure does not contain a readily-accessible functional handle for conjugation to various biomolecules necessary for targeting. Currently, there are no reports of Rhodamine 800 being used as a contrast agent for optoacoustic imaging. This may be due to its lack of an activated functional handle, or due to the overall lack of knowledge of optoacoustic signals for rhodamine dyes.



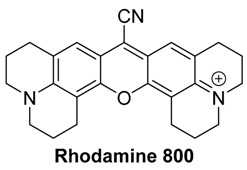



Unfortunately, in phantom studies, Rhodamine 800 did not perform as well as other commercially-available and often-utilized dyes. While the intensity of the absorbance spectrum of 10 µM of Rhodamine 800 measured on a spectrophotometer was comparable to other dyes at the same concentration, when phantoms containing the dye were imaged using MSOT, its optoacoustic signal was barely detectable. This makes it a less-than-ideal contrast agent for MSOT, especially when considering the need for imaging at depth, as the intensity would decrease even further.

### 2.2. Dyes with Absorbance > 720

Longer wavelength dyes with absorptions above 720 nm are typically green dyes, and many are based off of the cyanine scaffold. Of the far red dyes, only one is approved for human use by the FDA: indocyanine green (ICG). These compounds offer less background noise than the lower wavelength dyes, which enables their use at low concentrations. Due to this, they are often not detectable by the naked eye; however, fluorescence and optoacoustic imaging can produce strong signals.

#### 2.2.1. Indocyanine Green

Indocyanine green (ICG) is a heptamethine cyanine dye, and is one of the longest used NIR dyes for fluorescence imaging in medicine. It was approved for human use by the FDA in 1959, and has been widely used in medicine [71]. It has also seen use in optoacoustic imaging for numerous applications; however, it is a far-from-optimal probe. ICG suffers from poor water solubility, despite the charged sulfonate groups, and lacks a handle for covalent conjugation to targeting ligands, which prevents imaging of specific tissues. After IV injection, ICG remains in the blood with an average half-life of 3–5 min, and is filtered out largely by the liver (>97%) [72]. Due to its retention in blood and well-characterized pharmacokinetics, it has found use in ophthalmic angiography and pulmonary and hepatic function testing. Its highly-charged structure inhibits its use in any brain related imaging [73].



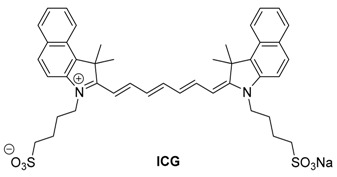



As one of the most well-characterized in vivo fluorescence imaging agents, ICG has seen a good amount of use in optoacoustic imaging as well. Buehler et al. have utilized ICG as a contrast agent for recording video rate images of kidney perfusion [74]. Using a custom-built ultrasound transducer array, they were able to obtain MSOT images of live animals at a 10 Hz frequency. Using this system, they were able to visualize the perfusion of ICG in mouse kidneys after tail vein injection, as shown in the representative MSOT images in Figure 16 (below).

**Figure 6:**
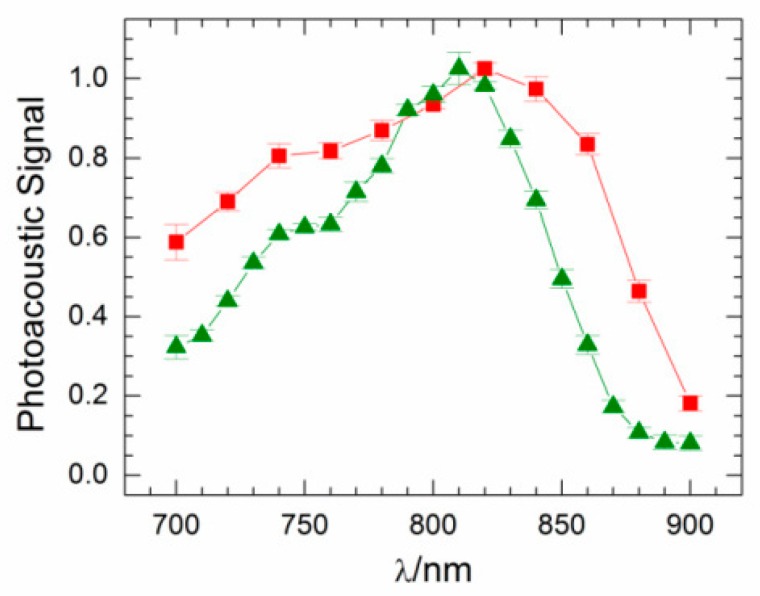
The optoacoustic spectra of free ICG in 3 μL/mL saline (■) and in 3 μL/mL plasma (▲). Eight trials were done, and the average of data for the various trials was plotted. Figure from [75] reproduced with permission from the publisher.

Wang et al. introduced an encapsulation method to traffic emulsion-based drug delivery [75]. ICG shows good optoacoustic signal in both saline solution and plasma. Notably, the ratio of the signals at 800 and 860 nm decreases by a factor of 2.6, going from saline solution to plasma (Figure 6) [75]. The authors attributed the difference to ICG binding to plasma protein [76]. They found that after ultrasound disruption of the encapsulation, an observable difference in optoacoustic signal was achieved.

**Figure 7:**
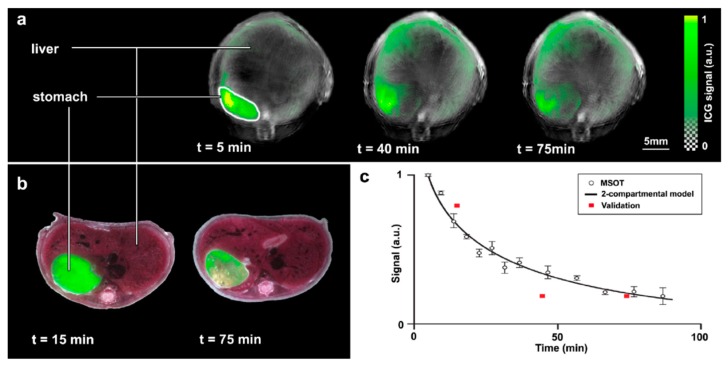
Gastric motility imaging using oral delivery of ICG. (**a**) ICG distribution after administration, (**b**) Ex vivo fluorescence verification in animals sacrificed after ICG dosing and (**c**) optoacoustic signal intensity over time compared with ex vivo fluorescence verification (red squares). Figure from [73] reproduced with permission from the publisher.

ICG has seen additional use for imaging several types of cancer as well as healthy tissues. In 2014, the Burton lab group investigated the use of ICG for monitoring medication-induced gastrointestinal intolerance [77]. Orally-dosed ICG was used to measure motility of the GI tract with MSOT shown in Figure 7.

Additionally, the optical properties of ICG have been reported to vary significantly with solvent choice, dye concentration, and environment [78,79]. While the intensity of the optoacoustic signal of ICG is proportional to the concentration of the dye, the shape of the spectrum can vary, which decreases its attractiveness of ICG as a contrast agent, especially as more dyes and targets are introduced [80]. This change in shape was observed in phantom studies with two different concentrations giving varying spectrums, which can be seen specifically with the loss of the left shoulder (Figure 16B). This modulation of spectral shape is not seen for the other dyes tested.

#### 2.2.2. IR780 Iodide

IR780 iodide is a cyanine dye similar to ICG, and features a maximum absorption at 780 nm. IR780 suffers from worse water solubility than ICG due to the lack of any charged groups in its basic structure. The central chlorine atom on IR780 permits substitutions to be made; however, multiple synthetic steps and purification are required to attach a targeting ligand [81]. IR780 also lacks FDA approval, so there is less literature for its use and safety. Nonetheless, IR780 and its derivatives have seen widespread use in the scientific community.



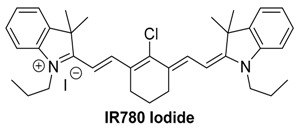



**Figure 8:**
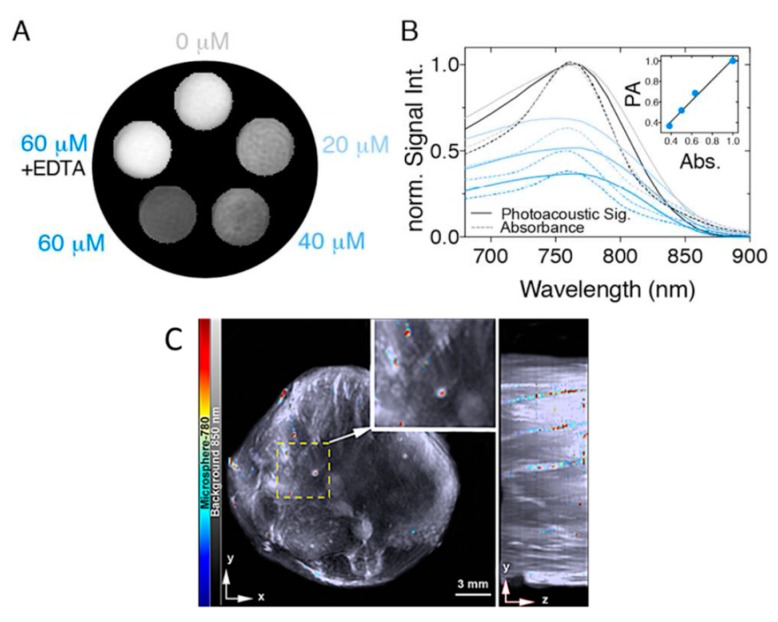
IR780-based probes have the potential to detect concentrations of calcium and serve as a tracking dye for monitoring microparticle accumulation. (**A**,**B**) Response of an IR780 based probe to calcium concentration. As calcium concentration increases, both absorbance and optoacoustic signal decrease. Figure from [82]. (**C**)—MSOT imaging of IR780 impregnated polystyrene microspheres in mouse liver after ileocolic vein injection. Figure from [83] reproduced with permission from the publisher.

IR780 is suitable for optoacoustic imaging due to its optical properties. With respect to maximizing optoacoustic signal, it is ideal that dyes have high molar absorptivity and low quantum yield. The rigidity of the polymethine chain of IR780 due to the cyclohexene ring allows the dye to have a high molar absorptivity and low quantum yield, making it desirable for optoacoustic imaging. Optimization of these compounds is a key feature in their utility, and the presence of the central chlorine allows for further development of this scaffold. Research done by the Westmyer group decorated IR780 with a calcium sensing functionality, which allowed optoacoustic tracking of calcium fluctuations [82]. In the presence of Ca^2+^, a hypochromic shift is observed on the absorption spectrum, and optoacoustic signal loss occurs. Binding of calcium was reversible, as the addition of EDTA to the probe/calcium solutions restored both the absorbance and optoacoustic signals (Figure 8A,B). Application of this probe is limited, however, by the inverse relation between signal and calcium concentration.

Additional reports exist of IR780 being used without synthetic modification by encapsulating the dye within nanostructures [83,84]. In 2017, Bhutiani et al. reported the detection of IR780 in mouse liver after polystyrene microsphere encapsulation. After injection into the ileocolic vein, the presence of microspheres in the liver was quantifiable using MSOT imaging (Figure 8C).

MSOT imaging of phantoms containing different concentrations of IR780 demonstrates the spectral stability with respect to concentration of the dye, as can be seen in Figure 8A,B. This spectral stability results in IR780 being a more attractive optoacoustic contrast agent than the related ICG for nanoparticle tracking.

#### 2.2.3. IRDye800 CW

Another heptamethine cyanine with a maximum absorption at 795 nm, IRDye800 CW contains multiple sulfonate groups, making it much more water soluble than ICG or IR780 iodide. Additionally, IRDye800 CW is sold with multiple different reactive groups (NHS ester, maleimide, azide, etc.), enabling facile conjugation to targeting ligands [85]. As with IR780 iodide, IRDye800 CW is not approved for human use by the FDA; however, due to the wide use of IRDye800 CW in the scientific literature, single-dose toxicity studies have been performed in rats, and 13 separate clinical trials for human use are ongoing [26,86].



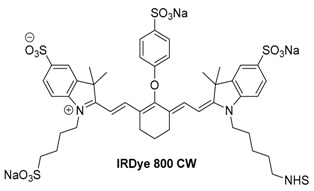



**Figure 9:**
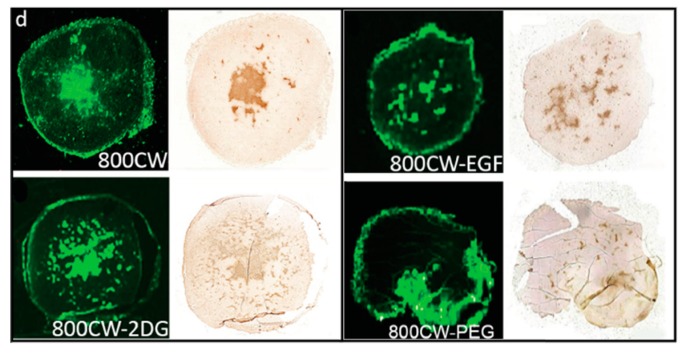
Imaging tumor necrosis with IRDye800 CW and its conjugates (2DG—2-deoxyglucose, EGF—endothelial growth factor, PEG—polyethylene glycol). IRDye 800 CW and its conjugates except 800CW-PEG showed co-localization with TUNEL staining which highlights damaged DNA. Figure from [90] reproduced with permission from the publisher.

IRDye 800 CW has seen widespread use in optoacoustic imaging due to its solubility and conjugatable handle. It has been used for kidney function testing, necrosis, and general and whole body imaging [87,88,89,90]. In 2015, the use of IRDye 800 CW and several of its conjugates was investigated for assessing tumor necrosis during chemotherapy as shown in Figure 9 [90].

It was found that while IRDye 800 CW and its conjugates were unable to penetrate the cell membrane of healthy or apoptotic cells, with the exception of the PEG-conjugated dye, all compounds showed selective uptake into necrotic cells with damaged membranes. Selective visualization of necrotic tissues would allow for improved tracking of chemotherapeutic efficacy during early stages of tumor treatment, when rapid evaluation of progression is key.

#### 2.2.4. Alexa Fluor 750

Alexa Fluor 750 is another heptamethine cyanine with a proprietary structure. Its maximal absorption is at 750 nm. It is available for purchase with several active groups for covalent conjugation to targeting ligands [91]. It is widely used in fluorescence imaging, and has seen a small amount use in optoacoustic imaging as well. Alexa Fluor has been used in imaging matrix metalloproteinase 2 and HER2+ breast cancer [92,93].

**Figure 10:**
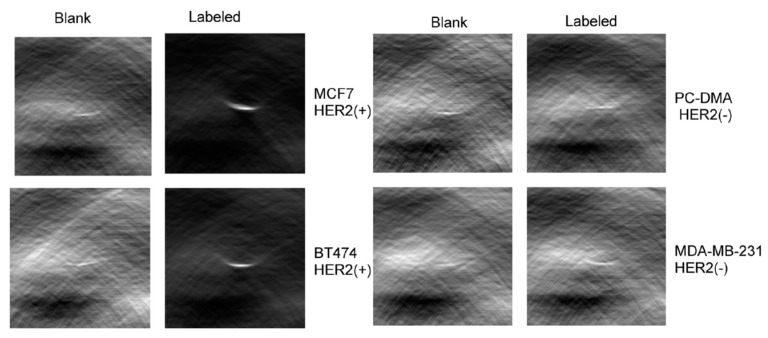
Imaging of HER2+ and HER2− cell lines with Alexa Fluor 750-Hercepin conjugates. Figure from [92] reproduced with permission from the publisher.

In 2008, Bhattacharyya et al. reported the use of Alexa Fluor 750 labeled Herceptin for imaging HER2+ cancer cell lines [92]. In in vitro testing, researchers were able to differentiate between HER2+ and HER2− cells based on the optoacoustic signal from the probe, as shown in Figure 10.

**Figure 11:**
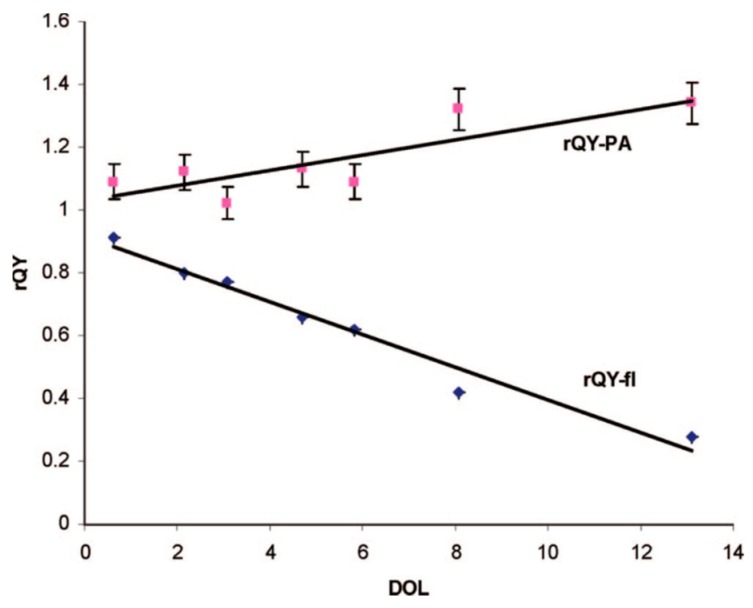
rQY dependence on degree-of-loading for Alexa Fluor 750-Hercepin conjugate. Figure from [92] reproduced with permission from the publisher.

Additionally, an interesting correlation was noted when testing the relative quantum yields (rQY) of both fluorescence and optoacoustic signal. They found that as the degree-of-labeling increased, the rQY of fluorescence decreased, while the rQY of optoacoustic signal increased proportionally as shown in Figure 11.

### 2.3. Quenchers

The majority of the exogenous contrast agents used for optoacoustic imaging have been fluorescent dyes. However, part of the energy absorbed by these dyes is emitted in the form of fluorescence, weakening the optoacoustic signal. Thus, for fluorescent dyes used for optoacoustic imaging, lower quantum yields are often desirable [94]. As an alternative, a group of dyes known as dark quenchers have the potential to be more ideal contrast agents. Dark quenchers emit the energy that was absorbed as heat instead of light. As heat is generated, so are sound waves, thus strengthening the optoacoustic signal [95]. Exploration of dark quenchers as exogenous contrast agents for optoacoustic imaging is still in its infancy. Only a few quenchers have been examined in vivo, and little is known about their interaction with other optoacoustically active dyes. While it is known that they can have a contact-quenching effect on a fluorescent probe’s absorbance spectrum [96], it is not known how or if they would have the same effect on optoacoustic signals from other exogenous dyes. Much is to be learned about their ability to be multiplexed.

#### 2.3.1. IRDye QC-1

IRDye QC-1 is a relatively new, non-fluorescent quenching dye. Quenchers such as this one are commonly used in combination with a fluorescent dye for FRET systems. FRET-based assays have been used to probe protease activity [97] as well as real-time monitoring of PCR [98]. Structurally, IRDye QC-1 is an unsymmetrical heptamethine cyanine dye with a dialkyl-amino substitution on the indole ring. It has been proposed that this amino substituent is the reason for the lack of fluorescence as well as the broadening of the absorbance spectra due to the lone pair of electrons on the nitrogen [99]. The use of multiple sulfonate groups again makes the dye somewhat water-soluble. QC-1 also comes equipped with an activated NHS-ester functional handle for conjugation to biomolecules of interest. In methanol, QC-1 has a very broad absorbance spectrum, with a peak at 737 nm. Because of its broad absorbance, QC-1 is an effective quencher for a wide range of NIR-emitting fluorescent dyes.



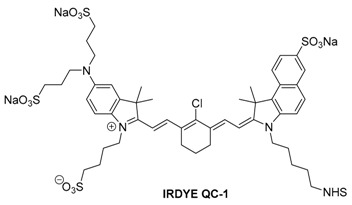



While the use of IRDye QC-1 as a contrast agent for optoacoustic imaging has not been reported in the literature yet, it has been presented as a viable dye at conferences.

In phantom studies, IRDye QC-1 gave a very strong, intense optoacoustic signal, but the spectrum was broad. This was in agreement with the absorbance spectrum. Additionally, it was observed that spectral shape obtained from MSOT imaging of dye-containing phantoms did not change significantly as the concentration of the dye was varied (Figure 16D).

#### 2.3.2. Black Hole Quencher 3 and QXL 680

Black Hole Quencher 3 (BHQ3) is a phenazinium-based dark quencher with a broad absorbance spectrum with peak absorption at 772 nm. In addition to FRET-based quenching, the BHQ series of quenchers are thought to quench fluorescence through contact-mediated quenching as well, which may give differing results to FRET-based quenchers [100].



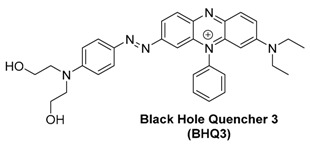



QXL 680 is another dark quencher optimized for Cy5 and Cy5.5 type dyes with maximum absorption at 679 nm [52]. Its structure is proprietary, so no information on how its quenching operates is known.

In a comparative study to develop activatable optoacoustic probes to detect overexpressed matrix metalloprotease 2 (MMP-2), BHQ3 and QXL 680, along with several dyes, were tested for their optoacoustic signaling potential [93]. Both quenchers gave a signal that was much stronger than any of the fluorescent dyes, and the final activatable probe was developed using the two quenchers without fluorescent dyes.

**Figure 12:**
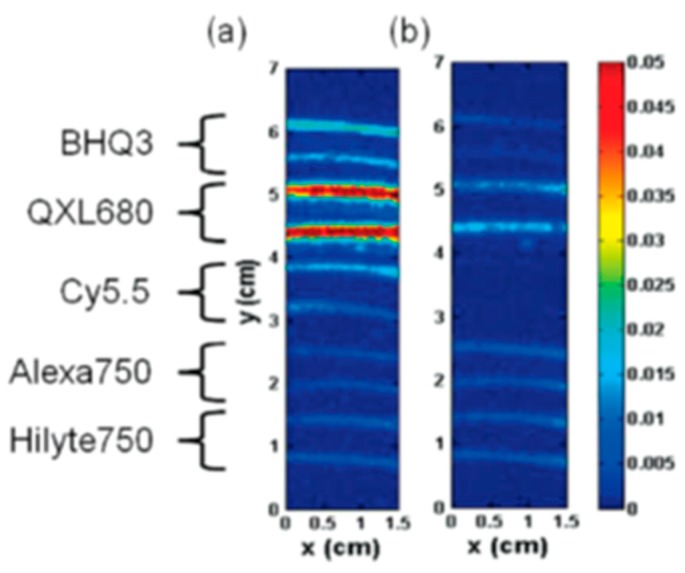
Relative optoacoustic signals from BHQ3, QXL680, and a variety of NIR dyes with 675 (**left**) and 750 nm (**right**) excitation. Figure from [93] reproduced with permission from the publisher.

**Figure 13:**
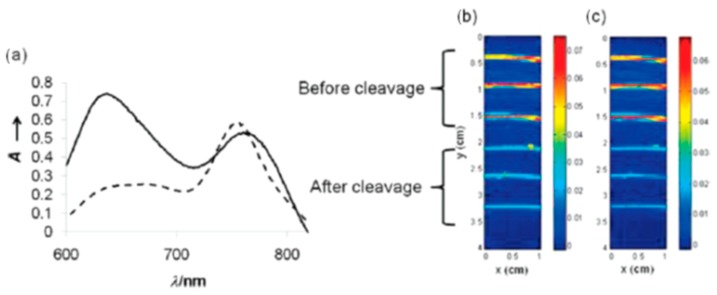
Quencher-based MMP-2 probe cleavage. (**a**) Absorbance spectra from before (solid line) and after (dashed line) MMP-2 activation of the probe. Optoacoustic imaging of probe before and after cleavage at 675 (**b**) and 750 nm (**c**). Figure from [93] reproduced with permission from the publisher.

Using the two quenchers, the researchers designed a probe which gave a clear signal change after enzymatic cleavage by MMP-2 as shown in Figure 12 and Figure 13.

In cell testing, the final probe gave a clearly differentiable signal for MMP positive cells versus the negative control. However, this probe has yet to move into animal imaging.

#### 2.3.3. QSY 21

QSY 21 is another dark quencher that has found use as an optoacoustic contrast agent. It has a peak absorption at 710 nm and an NHS ester for conjugation to targeting groups. It has been used in the noncovalent labeling of tumor-targeting single-walled carbon nanotubes (SWNTs) [101].



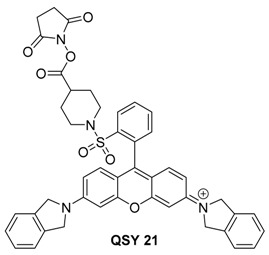



**Figure 14:**
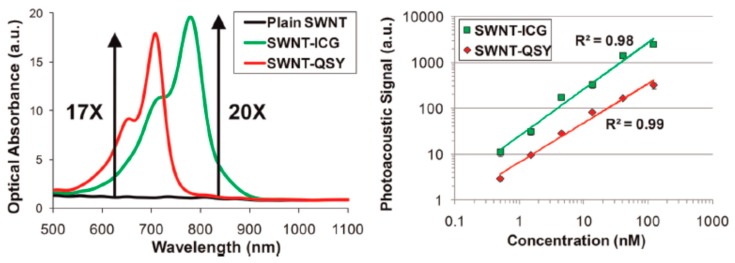
Comparison of absorbance (**left**) and optoacoustic signal (**right**) for ICG (green) and QSY 21 (red) labeled SWNTs. Figure from [101] reproduced with permission from the publisher.

De la Zerda et al. reported on SWNTs coated with small molecule dyes for use in multiplex imaging with a previously reported ICG labeled SWNT. Compared to the other tested dyes, QSY 21 gave a much stronger optoacoustic signal, and the peak absorption wavelength of 710 nm can be multiplexed with ICG (780 nm) as shown in Figure 14.

**Figure 15:**
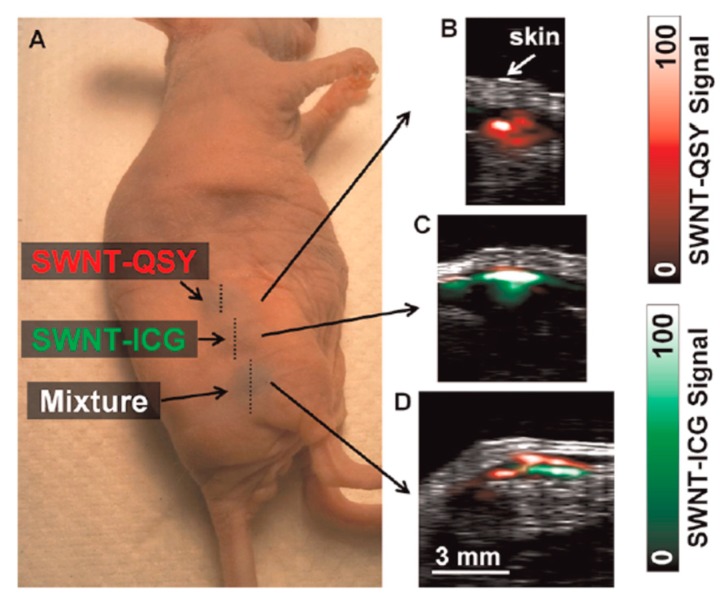
Animal testing of QSY 21 and ICG labeled SWNTs. Three separate subcutaneous injections were performed on a single mouse as shown in (**A**). The first contained QSY labeled SWNTs, the second contained ICG labeled SWNTS, and the third contained a mixture. These are shown respectively in inset images (**B**–**D**). Figure from [101] reproduced with permission from the publisher.

In animal testing, cRGD peptide-labeled SWNTs were found to target U87MG tumor xenografts. Moreover, spectral unmixing allowed for clear delineation of the two dyes, even in complex biological media as shown in Figure 15.

## 3. Summary and Outlook

**Figure 16:**
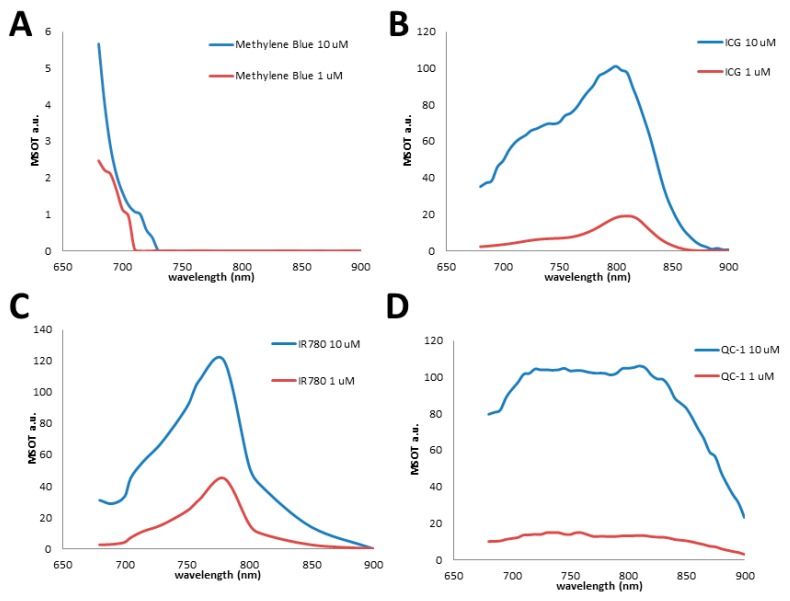
Optoacoustic spectral shapes of four representative dyes were evaluated at 1 µM (red line) and 10 µM (blue line) in tissue mimicking phantoms. Samples were measured at each 5 nm using an InVision 512-echo. (**A**) Methylene blue, (**B**) Indocyanine Green, (**C**) IR 780 iodide, (**D**) IRDye QC-1.

While there are a variety of commercially-available small molecule dyes that have shown promise as optoacoustic contrast agents, the need for more improved optoacoustic contrast agents is still great. One method used for developing fluorescent probes has been correlating physicochemical properties with in vivo behavior [102]. This method can be easily applied to optoacoustic imaging as well, and the physicochemical properties for the discussed commercially-available optoacoustic probes are shown in Table 1 and Table 2 below. Table 1 contains the directly-observable or experimentally-derived properties of the contrast agents, and Table 2 contains physicochemical properties calculated using ChemAxon software. It is often suggested that optimal optoacoustic probes possess the properties of a high molar extinction coefficient and a low fluorescent quantum yield [10]. It has also been speculated that the number of rotatable bonds may have an effect on the suitability of a probe for optoacoustic applications. Comparing the optoacoustic signal of methylene blue with ICG, IR780, and IRDye QC-1, methylene blue shows the lowest optoacoustic signal as well as the fewest rotatable bonds, lending some credence to this concept (Figure 16). Rhodamine 800, which showed no measurable optoacoustic response, likewise has no rotatable bonds. The difference could be explained by the formation of a twisted intramolecular charge transfer excited state for methylene blue [103]. Upon excitation, rotation of the amine groups and the formation of a biradicaloid species quenches the fluorescence of methylene blue. For rhodamine 800, the bridged cyclic amines are unable to rotate due to the strain this would put on the rings.

Within the last decade, there has been an enormous push to develop optoacoustic imaging that can be used clinically. With that push has come the need for small molecule dyes that are both optoacoustically active and well tolerated in vivo. Out of the dyes reviewed above, only three are FDA-approved for clinical use. An additional two are currently being tested in clinical trials; however, they are being evaluated for fluorescent applications, not for optoacoustic imaging [104]. Further, the large majority of the reviewed dyes absorb light under 800 nm, while currently-developed MSOT instruments deliver NIR light up to 950 nm. While there has been some development of fluorophores such as polymers and metal nanoparticles that absorb light between 800–950 nm, they often suffer from poor biocompatibility due to their lack of water solubility or their toxicity [100]. Again, this highlights the need for optoacoustically-active, small molecule probes that absorb in this region and which may be more amenable for biological imaging, and eventually, clinical use.

## 4. Materials and Methods for Optoacoustic Spectra in Figure 16

Dyes were diluted in distilled water at 1 µM and 10 µM respectively, and added to fixed cylindrical tissue mimicking phantoms of 2 cm diameter. Phantoms were prepared using a gel made from distilled water containing Agar (Sigma Aldrich, St. Louis, MO, USA) for jellification (1.3% *w*/*w*) and an intralipid 20% emulsion (Sigma Aldrich, St. Louis, MO, USA) for light diffusion (6% *v*/*v*), resulting in a gel presenting a reduced scattering coefficient of μ_s_ = 10 cm^−1^. Dyes were added to the 3 mm diameter cylindrical opening in the tissue phantoms [69,105]. All samples were evaluated using an inVision 512 MSOT (iTheraMedical, Munich, Germany) at each 5 nm between 680 nm–900 nm, as previously described [84,106]. Optoacoustic spectra were identified using the region of interest (ROI) method in a single slice and plotted in Figure 16.

## Figures and Tables

**Table 1:** Observable/Experimental physicochemical properties of selected dyes and quenchers.

	Molecular Weight	Rotatable Bonds	H-Bond Acceptors	Molar Absorptivity (M^−1^ cm^−1^)	Quantum Yield (%)
**DYES**					
ICG	751.97	10	6	183,000	3
MB	284.40	0	1	95,000	3
Evans Blue	868.85	4	20	7,810	N/A
IR780 iodide	540.20	4	0	274,000	7.6
IRDye800CW	1097.23	18	15	181,458	3.4
Rhodamine 800	396.50	0	2	113,302	25
Coomassie Blue	802.98	11	7	43,000	N/A
**QUENCHERS**					
IRDyeQC-1	1188.82	23	15	96,000	N/A
BHQ3	535.66	7	7	42,700	N/A
BlackBerry Quencher 650	576.60	6	12	41,000	N/A
QSY21	779.88	4	5	90,000	N/A

**Table 2:** Physicochemical properties of selected dyes and quenchers calculated using ChemAxon.

	Molecular Volume (Å^3^)	LogD	Surface Area (Å^2^)	TPSA (Å^2^)	Polorizability
**DYES**					
ICG	686.93	5.53	1143.82	181.76	87.37
MB	261.02	−0.62	388.00	43.91	32.74
Evans Blue	658.20	−3.62	987.04	396.09	81.28
IR780 iodide	536.10	6.67	898.85	50.60	66.43
IRDye800CW	920.12	−1.86	1536.72	385.83	108.94
Rhodamine 800	363.29	1.09	584.65	87.53	48.92
Coomassie Blue	716.81	4.54	1167.33	216.29	93.26
**QUENCHERS**					
IRDyeQC-1	1013.19	−0.69	1698.30	379.84	120.15
BHQ3	502.27	0.30	812.53	133.61	66.48
BlackBerry Quencher 650	503.94	0.94	828.01	157.09	61.17
QSY21	668.54	3.31	1055.44	173.18	89.10

LogD is calculated as an average of 3 methods.
